# Occupational Prevention of COVID-19 Among Healthcare Workers in Primary Healthcare Settings: Compliance and Perceived Effectiveness of Personal Protective Equipment

**DOI:** 10.1097/PTS.0000000000001004

**Published:** 2022-04-05

**Authors:** Muna Talal Theyab Abed Alah, Sami Abdeen, Nagah Selim, Elias Tayar, Iheb Bougmiza

**Affiliations:** From the ∗ Community Medicine Department, Hamad Medical Corporation (HMC); † Department of Family and Community Medicine, Primary Health Care Corporation, Doha, Qatar; ‡ Public Health and Preventive Medicine, Cairo University, Cairo, Egypt; § Community Medicine Department, Primary Health Care Corporation (PHCC), Doha, Qatar; ∥ Community Medicine Department, College of Medicine, Sousse University, Sousse, Tunisia.

**Keywords:** healthcare workers, compliance, personal protective equipment, COVID-19, primary health care, perceived effectiveness

## Abstract

It is crucial for healthcare workers (HCWs) to comply with infection prevention and control precautions such as the appropriate use of personal protective equipment (PPE) for their safety and the safety of patients. In this study, we aimed to assess HCWs’ compliance with the appropriate use of PPE in primary healthcare settings in Qatar along with its associated factors and explore their perceived effectiveness of different PPE items in protecting against COVID-19 infection.

**Methods:**

A Web-based survey was conducted between November 2020 and January 2021 targeting all clinical HCWs under the umbrella of Primary Health Care Corporation.

**Results:**

A total of 757 HCWs completed the survey, and most were between 30 and 39 years of age (50.2%), females (62.7%), and nurses (35.3%). Eighty eight percent of participants believed that PPE could provide high or very high protection against COVID-19. About one-half (53%) were found to be fully compliant with PPE use during patient interactions with suspected or confirmed COVID-19 cases, whereas three-quarters (76.3%) were fully compliant while performing aerosol-generating procedures. Healthcare workers’ age, nationality, health center region, area of work, clinical experience, frequency of interaction with suspected or confirmed COVID-19 cases, and the perceived effectiveness of PPE were significant predictors of full compliance with PPE. Shortage of PPE was the commonest reported barrier to appropriate use.

**Conclusions:**

Despite HCWs’ high perceived effectiveness for PPE in protecting against COVID-19 infection, their full compliance rate with using PPE was moderate and needs further improvement.

Infectious outbreaks can overwhelm health systems and put healthcare workers (HCWs) at increased risk of infection while dealing with increasing numbers of infected cases.^1^ The current severe acute respiratory syndrome coronavirus 2 (SARS-CoV-2) has infected more than 50% of HCWs in several countries.^1^ Beside the importance of practicing hand hygiene, HCWs need to strictly follow the World Health Organization (WHO) recommendations by using droplet and contact precautions (including the use of a medical mask; eye protection [goggles] or facial protection [face shield]; a clean, nonsterile, long-sleeved gown; and gloves) during regular patient interactions with suspected or confirmed COVID-19 cases, and airborne precautions using N95 respirator or equivalent along with contact precautions while performing aerosol-generating procedures (AGPs).^2^ The evolving literature has shown variable rates of compliance with personal protective equipment (PPE) among HCWs during the current COVID-19 pandemic. Where only 31.5%, 46.8%, and 54.9% of HCWs were consistently compliant with PPE in Brazil, Egypt, and Congo, respectively, compliance rates exceeded 80% in each of Germany and Ghana.^3–7^ Healthcare-associated infections resulting from low compliance with infection prevention and control precautions can have negative repercussions on HCWs, patients, and health systems resulting in prolonged hospital stays and adverse health and economic consequences.^8–11^ Several factors affect HCWs’ compliance with PPE such as the availability of PPE, seniors compliance, past experience, training on the appropriate use of PPE, discomfort caused by certain types of PPE, performing high COVID-19 risk procedures, and the perceived effectiveness of PPE.^3,12,13^ Evidence has shown that the high perceived effectiveness of PPE is a strong predictor for high compliance.^13,14^

In Qatar, among HCWs who were tested for COVID-19, 10.6% and 16.2% tested positive in secondary and primary healthcare settings, respectively.^15,16^ Primary Health Care Corporation (PHCC)—the main provider of primary healthcare services in Qatar—has played a crucial and proactive role in mitigating the spread of COVID-19 infection by converting some of the health centers into COVID-19 facilities, assisting in contact tracing, and early detection of cases by dedicating some of their healthcare staff for the provision of drive-through swabbing.^16^ In fact, starting in March 2020, PHCC dedicated a number of health centers as testing and holding facilities for COVID-19, whereas some rearrangements were made to other health centers to separate suspected cases of COVID-19 from other patients.^17^ At the time of conducting this study, PHCC was providing its services through 27 health centers distributed all over Qatar (7 health centers in the central region and 10 in each of the western and northern regions). Although telephone consultations have replaced face-to-face consultations in many routine clinical settings, some primary healthcare services continued operating regularly with face-to-face consultations such as childhood immunization services (Well Baby clinic); antenatal clinics for pregnant women, which provided both types of consultations according to trimesters; emergency department; and laboratory and diagnostic radiology services. In this study, we aimed to assess the compliance with the appropriate use of PPE among HCWs in primary healthcare settings in Qatar along with its associated factors and explore HCWs’ perceived effectiveness of different PPE items in protecting against COVID-19 infection.

## METHODS

### Study Design, Setting, and the Target Population

We conducted a Web-based cross-sectional study between November 2020 and January 2021 targeting all clinical staff (physicians, nurses, and allied health professionals) working under the umbrella of PHCC. Primary Health Care Corporation is the state-owned primary healthcare provider in Qatar that provides primary healthcare services through a large number of healthcare centers distributed all over the country. According to the annual statistical report by PHCC, the total staff amounted to 6631 employees in 2019, with 4429 (67%) of them being clinical staff.^18^

### Study Procedure

We developed an online self-administered survey using Google Forms. Because the response rate in Web-based surveys is generally low, we invited all eligible HCWs in clinical positions in PHCC. We contacted HCWs via email with a link to the online survey. We started the survey with an introductory letter that explained the objectives of the study and ensured the voluntary participation, the anonymity, and the confidentiality of the collected data. Taking the survey implied informed consent, and the participants had the option to quit the survey at any time. Reminders were sent regularly. We obtained the ethical approval from the institutional review board of PHCC (PHCC/DCR/2020/07/073). This study followed the Strengthening the Reporting of Observational Studies in Epidemiology (STROBE) checklist for cross-sectional studies.

### Study Questionnaire

The questionnaire was adopted from different surveys.^19–23^ It consisted of 3 sections. The first one explored the sociodemographic characteristics of the participants (age, sex, nationality, profession, clinical experience, healthcare facility [whether a COVID-19 facility or not], area of work, the name of the health center) and some background information such as having a friend or a relative infected with COVID-19, frequency of interaction with suspected or confirmed COVID-19 cases, and training on PPE. The second and third sections assessed the perceived effectiveness of different PPE items in protecting against COVID-19 infection and compliance with the appropriate use of PPE using the WHO risk assessment tool for HCWs in the context of COVID-19,^23^ along with the barriers to the appropriate use, respectively.

### Study Variables

We assessed HCWs’ perceived effectiveness of different PPE items in protecting against COVID-19 by asking them to indicate their degree of agreement with a set of statements on a 4-point Likert scale. The 4 points on the scale were as follows: 1 (strongly disagree), 2 (disagree), 3 (agree), and 4 (strongly agree). Examples of such statements were as follows: “I believe that regular face mask (medical or surgical) is effective and can help against contracting COVID-19 infection,” “I believe that gloves are effective and can help against contracting COVID-19 infection,” and so on. Also, we asked HCWs to indicate the degree of protection PPE generally can provide on a 5-point Likert scale. The points on the scale were 1 (no or very low protection), 2 (low protection), 3 (moderate protection), 4 (high protection), and 5 (very high protection). We calculated a perceived effectiveness score by summing the points for all statements with a maximum score of 25. Higher scores indicated higher perceived effectiveness of PPE in protecting against contracting COVID-19. We assessed HCWs’ compliance with PPE by asking them to state the frequency of using different PPE items during regular interactions with suspected or confirmed cases or while performing an AGP for them using a 5-point Likert scale (always as recommended, often, sometimes, seldom, never). We considered those who answered all the questions as “always as recommended” as fully compliant, and they were counted in the calculation of the proportions of HCWs who were fully compliant with PPE in different settings (whether during regular patient interactions, while performing AGPs, or during both situations). We asked HCWs to select the barriers to the appropriate PPE use from a list, and they had the option to disclose other barriers that were not listed.

### Statistical Analysis

We carried out the data analysis using IBM SPSS Statistics for Windows, version 26.0 (IBM, Armonk, NY). We presented descriptive statistics as frequencies and percentages for categorical variables, and as medians and interquartile ranges for continuous not normally distributed variables. We used the χ^2^ test to determine the differences in compliance rates between different groups. After testing for normality using the Shapiro-Wilk test, we used Mann-Whitney *U* and Kruskal-Wallis tests to compare perceived effectiveness scores among different groups. We carried out a multivariable logistic regression model to determine the predictors of full compliance with PPE in different settings (during both regular patient interactions with suspected or confirmed COVID-19 cases, and while performing an AGPs). The associations between predictors and outcomes were presented as adjusted odds ratios (ORs) and 95% confidence intervals (95% CIs). We assessed the goodness of fit using the Hosmer-Lemeshow test. *P* values of less than 0.05 were considered significant.

### Ethical Approval

This study was performed in line with the principals of Declaration of Helsinki. Ethical approval was obtained from PHCC under protocol ID PHCC/DCR/2020/07/073.

## RESULTS

### Sociodemographic Characteristics and Background Information

A total of 757 HCWs completed the survey. Most, 380 (50.2%), were between 30 and 39 years of age, and 475 (62.7%) were females. With more than 60 nationalities reported, the top 3 were Filipino (26.9%), Indian (25.1%), and Egyptian (11.9%). Of all HCWs, 267 (35.3%) were nurses, 150 (19.8%) were physicians, and the remaining were other health professionals like dentists, allied HCWs, pharmacists, and others. Most HCWs, 302 (39.9%), were from health centers in the western region, and 368 (48.6%) had a clinical experience of 5 years or more. About one-fifth (19.9%) of participants were deployed to a COVID-19 facility. More than half of the participants (58.8%) reported frequent dealing with suspected or confirmed COVID-19 cases in most of the shifts or every shift, and most of HCWs (81.2%) were aware of a relative or a friend diagnosed with COVID-19. Upon assessing the status of training on appropriate PPE use in the previous year, 88.2% admitted receiving such training (Table 1).

**TABLE 1:** Sociodemographic Characteristics and Related Background Information of the Participants by the Type of Profession

Characteristic	Profession			Total (N = 757), No. (%)	*χ*^2^ Test, *P* Value
	Nurse (n = 267), No. (%)	Physician (n = 150), No. (%)	Others (n = 340), No. (%)		
Age category, y					
18–29	28 (10.5)	0 (0.0)	38 (11.2)	66 (8.7)	**<0.001**
30–39	179 (67.0)	39 (26.0)	162 (47.6)	380 (50.2)	
40–49	43 (16.1)	64 (42.7)	105 (30.9)	212 (28.0)
≥50	17 (6.4)	47 (31.3)	35 (10.3)	99 (13.1)
Sex					
Female	234 (87.6)	60 (40.0)	181 (53.2)	475 (62.7)	**<0.001**
Male	33 (12.4)	90 (60.0)	159 (46.8)	282 (37.3)	
Nationality*					
Indian	112 (41.9)	10 (6.7)	68 (20.0)	190 (25.1)	**<0.001**
Filipino	96 (36.0)	2 (1.3)	106 (31.2)	204 (26.9)	
Egyptian	20 (7.5)	29 (19.3)	41 (12.)	90 (11.9)	
Jordanian	11 (4.1)	17 (11.3)	34 (10.0)	62 (8.2)	
Sudanese	1 (0.4)	7 (4.7)	45 (13.2)	53 (7.0)	
Others	27 (10.1)	85 (56.7)	46 (13.5)	158 (20.9)	
PHCC region					
Northern	99 (37.1)	52 (34.7)	142 (51.8)	293 (38.7)	0.520
Central	62 (23.2)	32 (21.3)	68 (20.0)	162 (21.4)	
Western	106 (39.7)	66 (44.0)	130 (38.2)	302 (39.9)	
Area of work					
Non–COVID-19 facility					
Clinic	136 (50.9)	108 (72.0)	145 (42.6)	389 (51.4)	**<0.001**
Emergency					
	17 (6.4)	7 (4.7)	2 (0.6)	26 (3.4)	
Others	47 (17.6)	6 (4.0)	138 (40.6)	191 (25.2)	
COVID-19 facility	67 (25.1)	29 (19.3)	55 (16.2)	151 (19.9)	
Clinical experience (in PHCC), y					
<1	50 (18.7)	24 (16.0)	36 (10.6)	110 (14.5)	**0.004**
1–2	47 (17.6)	30 (20.0)	57 (16.8)	134 (17.7)	
3–4	45 (16.9)	17 (11.3)	83 (24.4)	145 (19.2)	
≥5	125 (46.8)	79 (52.7)	164 (48.2)	368 (48.6)	
Frequency of dealing with COVID-19 suspected or confirmed cases during clinical practice					
Never	9 (3.4)	22 (14.7)	55 (16.2)	86 (11.4)	**<0.001**
Some of my shifts	47 (17.6)	51 (34.0)	128 (37.6)	226 (29.9)	
Most of my shifts	40 (15.0)	17 (11.3)	42 (12.4)	99 (13.1)	
Every shift	171 (64.0)	60 (40.0)	115 (33.8)	346 (45.7)	
Having a relative or friend diagnosed with COVID-19					
No	43 (16.1)	28 (18.7)	71 (20.9)	142 (18.8)	0.326
Yes	224 (83.9)	122 (81.3)	269 (79.1)	615 (81.2)	
Training on proper use of PPE in the past year					
No	9 (3.4)	16 (10.7)	64 (18.8)	89 (11.8)	**<0.001**
Yes	258 (96.6)	134 (89.3)	276 (81.2)	668 (88.2)	

Significant *P* values of <0.05 are shown in bold.

*More than 60 nationalities were reported.

### HCWs’ Perception of Effectiveness of Different PPE Items Against COVID-19 Infection

Of all HCWs, 83%, 97.8%, 95.4%, 94.7%, and 92.4% agreed or strongly agreed that regular face mask (medical or surgical), respirator, eye protection (using goggles or a face shield), long-sleeved gown, and gloves are effective in protecting against contracting COVID-19 infection, respectively. Most participants, 666 (88%), believed that PPE could provide high or very high protection against COVID-19. The calculated median of the perceived effectiveness score was 22, with 406 (53.6%) scored ≥22. The uni-variable analysis showed a statistically significant difference in the perceived effectiveness scores between HCWs of different nationalities and different areas of work. Healthcare workers of Filipino nationality reported significantly higher perceived effectiveness scores compared with all other nationalities (*P* < 0.001). Similarly, higher proportions of HCWs with higher perceived effectiveness scores were found in COVID-19 facilities compared with non–COVID-19 facilities (*P* = 0.013). Moreover, participants who received a training on appropriate PPE use in the previous year had higher scores compared with those who did not (*P* = 0.010; Table 2).

**TABLE 2:** Differences in the Perceived Effectiveness Score for PPE Among Different Subgroups

Variable	Perceived Effectiveness Score, Median (IQR)	P*
Age category, y		
18–29	21 (19–23)	0.343
30–39	22 (19–24)	
40–49	22 (19–24)	
≥50	22 (19–24)	
Sex		
Female	22 (19–24)	0.088
Male	22 (19–24)	
Nationality^†^		
Filipino	23 (21–24)	**<0.001**
Indian	22 (19–24)	
Egyptian	21 (19–23)	
Jordanian	20 (19–23)	
Sudanese	21 (19–23)	
Others	21 (19–24)	
PHCC region		
Northern	22 (19–24)	0.136
Central	21 (19–24)	
Western	22 (19–24)	
Area of work^‡^		
Non–COVID-19 facility		
Clinic	22 (19–24)	**0.013**
Emergency	23 (20–24)	
Others	21 (19–24)	
COVID-19 facility	23 (20–24)	
Clinical experience (in PHCC), y		
<1	22 (19–24)	0.706
1–2	22 (19–24)	
3–4	22 (19–24)	
≥5	22 (19–24)	
Profession		
Physician	22 (19–24)	0.539
Nurse	22 (19–24)	
Others	22 (19–24)	
Having a relative or friend diagnosed with COVID-19		
No	22 (19–24)	0.500
Yes	22 (19–24)	
Training on the proper use of PPE in the past year		
No	19 (18–21)	**0.010**
Yes	22 (19–24)	

Significant *P* values of <0.05 are shown in bold.

*Using the Mann-Whitney test for 2 independent samples and the Kruskal-Wallis test for 3 or more independent samples.

^†^Pairwise comparisons showed statistically significant differences between each of Indian, Egyptian, Jordanian, Sudanese, and other nationalities compared with Filipino nationality (*P* = 0.002, 0.001, 0.005, 0.002, and <0.001, respectively).

^‡^Pairwise comparisons showed statistically significant differences between each of those who worked in clinic or other non–COVID-19 facilities compared with those who worked in COVID-19 facility (*P* = 0.038 and 0.009, respectively).

IQR, interquartile range.

### HCWs’ Compliance With PPE

**FIGURE 1:**
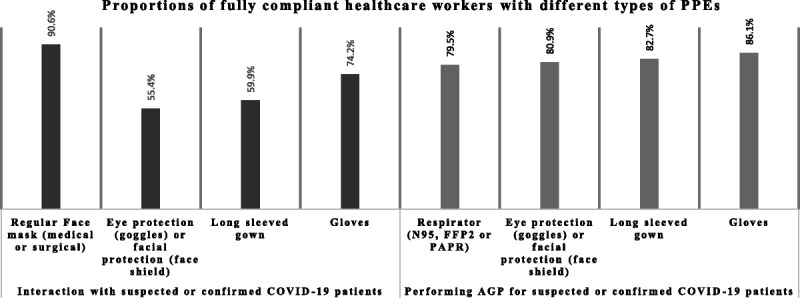
Proportions of fully compliant HCWs with different types of PPEs in different situations.

We assessed the compliance of HCWs with PPE using the WHO risk assessment tool for HCWs in the context of COVID-19.^23^ Using this tool, 328 of 619 (53%; 95% CI, 49.0–57.0; who deal with suspected or confirmed COVID-19 cases) were found to be fully compliant with PPE use during patient interactions with suspected or confirmed COVID-19 cases, 384 of 503 (76.3%; 95% CI, 72.4–80.0; who perform AGPs for suspected or confirmed COVID-19 cases) were fully compliant with PPE use while performing such procedures, and 257 (51.1%; 95% CI, 46.6–55.5) were fully compliant during both situations. During routine patient interactions with suspected or confirmed cases, full compliance rates were found to be highest with face mask (90.6%) and lowest with eye protection (using goggles) or facial protection (using a face shield; 55.4%). On the other hand, we could not detect such a gap in the full compliance rates between different PPE items while performing AGPs for suspected or confirmed COVID-19 cases (Fig. 1).

The univariable analysis showed that nationality, area of work, profession, frequency of dealing with suspected or confirmed COVID-19 cases, and the perceived effectiveness of PPE were significantly associated with full compliance (Table 3).

**TABLE 3:** Determinants and Predictors of Full Compliance With PPE Measures Using χ^2^ Test and Multiple Logistic Regression Analysis

Variable	PPE Compliance*			
	Fully Compliant, No. (%)^†^	χ^2^ Test, *P* Value	Multivariable Regression Analysis	
			AOR (95% CI)	*P*
Age category, y				
18–29	18 (7.0)	0.138	0.21 (0.07–0.61)	**0.004**
30–39	135 (52.5)		0.39 (0.18–0.82)	**0.013**
40–49	70 (27.2)		0.49 (0.24–1.01)	0.054
≥50	34 (13.2)		1 (reference)	
Sex				
Female	160 (62.3)	0.914	0.997 (0.63–1.58)	0.991
Male	97 (37.7)		1 (reference)	
Nationality				
Filipino	85 (33.1)	**0.032**	1 (reference)	
Indian	68 (26.5)		0.65 (0.38–1.12)	0.121
Egyptian	23 (8.9)		0.51 (0.24–1.11)	0.089
Jordanian	16 (6.2)		0.47 (0.20–1.11)	0.085
Sudanese	8 (3.1)		0.31 (0.11–0.84)	**0.021**
Others	57 (22.2)		0.50 (0.25–1.01)	0.052
PHCC region				
Northern	97 (37.7)	0.464	0.88 (0.57–1.37)	0.576
Central	48 (18.7)		0.55 (0.32–0.93)	**0.026**
Western	112 (43.6)		1 (reference)	
Area of work				
Non–COVID-19 facility				
Clinic	149 (58.0)	**0.039**	1 (reference)	
Emergency	12 (4.7)		0.76 (0.28–2.06)	0.588
Others	38 (14.8)		0.60 (0.34–1.04)	0.069
COVID-19 facility	58 (22.6)		0.47 (0.28–0.79)	**0.004**
Profession				
Nurse	115 (44.7)	**0.025**	1 (reference)	
Physician	63 (24.5)		1.25 (0.62–2.52)	0.542
Others	79 (30.7)		0.80 (0.47–1.35)	0.400
Clinical experience (in PHCC), y				
<1	49 (19.1)	0.446	2.45 (1.25–4.79)	**0.009**
1–2	42 (16.3)		1.22 (0.65–2.26)	0.535
3–4	50 (19.5)		1.23 (0.70–2.16)	0.470
≥5	116 (45.1)		1 (reference)	
Having a relative or friend diagnosed with COVID-19				
No	49 (19.1)	0.483	1.41 (0.84–2.36)	0.189
Yes	208 (18.9)		1 (reference)	
Frequency of interaction with COVID-19 suspected or confirmed patients				
Never	17 (6.6)	**0.030**	1.20 (0.47–3.05)	0.697
Some of the shifts	56 (21.8)		0.54 (0.32–0.90)	**0.018**
Most of the shifts	33 (12.8)		0.52 (0.30–0.92)	**0.025**
Every shift	151 (58.8)		1 (reference)	
Training on the proper use of PPE in the past year				
No	19 (7.4)	0.124	0.84 (0.41–1.72)	0.639
Yes	238 (92.6)		1 (reference)	
Perceived effectiveness category				
Low perception	84 (32.7)	**<0.001**	1 (reference)	
High perception	173 (67.3)		2.48 (1.66–3.70)	**<0.001**

Significant *P* values of <0.05 are shown in bold.

*The outcome of the regression model is compliance with PPE (N = 503) including compliance during interaction with suspected or confirmed COVID-19 cases and while performing an AGP for suspected or confirmed COVID-19 case.

^†^Because of rounding, percentages may not always appear to add up to 100%.

AOR, adjusted OR.

### Predictors of HCWs’ Compliance With PPE

As shown in Table 3, we carried out a multivariable logistic regression analysis to determine the predictors of full compliance with PPE in different settings (during both regular patient interactions with suspected or confirmed COVID-19 cases, and while performing an AGP for a suspected or confirmed case). The model was of good fit and statistically significant (*χ*^2^_25_ = 76.607, *P* < 0.001) when compared with the null model. As shown in Table 3, age, nationality, PHCC region, area of work, clinical experience, frequency of interaction with suspected or confirmed COVID-19 cases, and the perceived effectiveness of PPE were found to be significantly and independently associated with full compliance with PPE. Healthcare workers 18 to 29 years of age (OR, 0.21; 95% CI, 0.07–0.61; *P* = 0.004) and those 30 to 39 years of age (OR, 0.39; 95% CI, 0.18–0.82; *P* = 0.013) were less likely to be fully compliant with PPE compared with those 50 years or older. Sudanese HCWs were less likely to be fully compliant compared with Filipino HCWs (OR, 0.31; 95% CI, 0.11–0.84; *P* = 0.021). Healthcare workers from health centers at the central region were less likely to be fully compliant than those from health centers at the western region (OR, 0.55; 95% CI, 0.32–0.93; *P* = 0.026). We found that the participants who were working in COVID-19 facilities were less likely to be fully complaint compared with those running primary healthcare clinics at non–COVID-19 facilities (OR, 0.47; 95% CI, 0.28–0.79; *P* = 0.004). Healthcare workers who deal with suspected or confirmed COVID-19 cases on some of the shifts (OR, 0.54; 95% CI, 0.32–0.90; *P* = 0.018) or most of shifts (OR, 0.52; 95% CI, 0.30–0.92; *P* = 0.025) were less likely to be fully compliant compared with those who deal with such cases on every shift. On the other hand, HCWs with less than 1 year of experience and those with high perceived effectiveness scores (≥22) of PPE were more than 2 times more likely to be fully complaint compared with those with 5 or more years of experience and those with low perceived effectiveness scores (<22), with an OR of 2.45 (95% CI, 1.25–4.79; *P* = 0.009) and an OR of 2.48 (95% CI, 1.66–3.70; *P* < 0.001), respectively.

### Barriers to the Appropriate Use of PPE

Upon assessing the barriers to the appropriate use of PPE, the top 3 barriers were shortage of PPE (42.3%), discomfort caused by certain types of PPE such as face mask or face shield (31.4%), and work overload and lack of time (29.3%). On the other hand, 29.5% of participants did not report any barriers for the appropriate use of PPE, whereas shortage of PPE was the most common reported barrier among all HCWs in different areas of work, professions, and health centers regions (Table 4).

**TABLE 4:** Barriers to the Proper Use of PPE by PHCC Regions, Profession, and Area or Work

PPE Barriers	PHCC Regions			Profession			Area of Work				Total, No. (%)
	Northern, No. (%)	Central, No. (%)	Western, No. (%)	Nurse, No. (%)	Physician, No. (%)	Others, No. (%)	COVID19 Facility, No. (%)	Non–COVID-19 Facility			
								Clinic, No. (%)	Emergency, No. (%)	Others, No. (%)	
Shortage of PPE	118 (40.3)	70 (43.2)	132 (43.7)	117 (43.8)	64 (42.7)	139 (40.9)	70 (46.4)	169 (43.4)	8 (30.8)	73 (38.2)	320 (42.3)
Work overload and lack of time	84 (28.7)	41 (25.3)	97 (32.1)	82 (30.7)	46 (30.7)	94 (27.6)	30 (19.9)	126 (32.4)	11 (42.3)	55 (28.8)	222 (29.3)
Forgetfulness	22 (7.5)	12 (7.4)	23 (7.6)	14 (5.2)	13 (8.7)	30 (8.8)	5 (3.3)	25 (6.4)	5 (19.2)	22 (11.5)	57 (7.5)
Lack of knowledge of proper use of PPE	19 (6.5)	14 (8.6)	34 (11.3)	20 (7.5)	7 (4.7)	40 (11.8)	11 (7.3)	37 (9.5)	1 (3.8)	18 (9.4)	67 (8.9)
Low COVID-19 risk perception	18 (6.1)	9 (5.6)	14 (4.6)	11 (4.1)	3 (2.0)	27 (7.9)	9 (6.0)	19 (4.9)	0 (0.0)	13 (6.8)	41 (5.4)
Low perceived effectiveness of PPE	14 (4.8)	11 (6.8)	14 (4.6)	10 (3.7)	4 (2.7)	25 (7.4)	8 (5.3)	22 (5.7)	1 (3.8)	8 (4.2)	39 (5.2)
Discomfort caused by PPE	81 (27.8)	52 (32.1)	105 (34.8)	80 (30.0)	49 (32.7)	109 (32.1)	51 (33.8)	116 (29.8)	7 (26.9)	64 (33.5)	238 (31.4)
Interference of patient-provider relationship	31 (10.6)	17 (10.5)	27 (8.9)	28 (10.5)	8 (5.3)	39 (11.5)	14 (9.3)	35 (9.0)	1 (3.8)	25 (13.1)	75 (9.9)
Lack of policies/sanctions/penalties for noncompliers	12 (4.1)	9 (5.6)	18 (6.0)	15 (5.6)	5 (3.3)	19 (5.6)	10 (6.6)	19 (4.9)	1 (3.8)	9 (4.7)	39 (5.2)
Lack of PPE training	22 (7.5)	14 (8.6)	28 (9.8)	18 (6.7)	10 (6.7)	36 (10.6)	15 (9.9)	30 (7.7)	1 (3.8)	18 (9.4)	64 (8.5)
Noncompliance of colleagues and supervisors (peer effect)	17 (5.8)	12 (7.4)	10 (3.3	11 (4.1)	7 (4.7)	21 (6.2)	8 (5.3)	22 (5.7)	0 (0.0)	9 (4.7)	39 (5.2)

## DISCUSSION

Healthcare workers are among the frontliners in the fight against COVID-19, and thus, they are at a high risk of contracting the infection. The nature of their work, which involves direct, close, and prolonged contact with patients, has placed them among the high-risk groups for acquiring the infection during the COVID-19 pandemic.^24^ In this study, we assessed HCWs’ compliance with PPE and their perceived effectiveness of different PPE items. We found that more than 80% of HCWs believed in the effectiveness of PPE in protecting them against contracting COVID-19 infection. However, they perceived that certain types of PPE are superior to others in preventing the spread of the infection, particularly when it comes to face masks and respirators, where 83%, and 97.8% of HCWs found them effective, respectively. Consisting with a finding reported in a study conducted during the SARS outbreak.^19^ Healthcare workers working in COVID-19 facilities had significantly higher perceived effectiveness scores than those working in non–COVID-19 facilities. One explanation might be that HCWs in COVID-19 facilities are probably more aware about the infection, transmission modes, and the actual effectiveness of different types of PPE, which we believe might affect their perception of the effectiveness of PPE in protecting against COVID-19. In addition, during their work in such facilities, they might have witnessed cases that turned to be positive because of inconsistent use of PPE whether in the work environment or among the public, which might also influenced their perception. Regarding HCWs’ compliance with PPE, some studies that used the same assessment tool (WHO checklist) demonstrated similar compliance rates in Congo (54.9%) and higher rates in Ghana (90.6%).^5,7^ On the other hand, compliance rate in our study was higher than what was reported in a study in Brazil (31.5%).^4^ Such variability in rates could be related to the time of conducting the studies in relation to the phase of the pandemic in each country, the level of HCWs’ awareness of the importance of complying with PPE, COVID-19 risk perception among HCWs in different countries, and the perceived effectiveness of PPE in protecting against contracting the infection. The lowest compliance rates during patient interactions were found with the use of eye protection (using goggles) or facial protection (using a face shield), which is similar to a study conducted in Egypt.^3^ This might be attributed to the discomfort caused by these PPE items. One study in China that assessed the discomfort caused by different types of PPE during COVID-19 pandemic demonstrated a comparatively high occurrence of all the types of discomfort, which were reported by more than 40% of HCWs, with the top complaints being retroauricular pain (mask pressure related), chest distress or dyspnea, and inconvenience at work, followed by thirst or dry throat.^25^ Moreover, in our study, we found that the discomfort caused by certain types of PPE like face mask, goggles, or face shield is among the top barriers to the compliance with PPE. Several measures can be followed to minimize the discomfort caused by PPE and improve compliance. For example, the use of a dressing can decrease the chance of incurring pressure injuries that can be caused by certain types of PPE in certain areas such as the bridge of the nose and behind the ears, using a headband with buttons that attach to the straps of a surgical mask, wearing appropriate size of PPE, and removing PPE as soon as possible after leaving the work environment.^26,27^ Having younger age groups less compliant with PPE might be attributed to their knowledge of the fact that the risk and severity of COVID-19 increase in older adults starting in their 50s and in those with chronic medical conditions who are usually of older age.^28^ Surprisingly, despite having higher perceived effectiveness, participants who were working in COVID-19 facilities were less likely to be fully complaint compared with those running primary healthcare clinics at non–COVID-19 facilities, which might be attributed to the shortage of PPE as reported by more HCWs among COVID-19 facilities compared with non–COVID-19 facilities, which is expected in light of the large number of suspected COVID-19 cases dealt with in COVID-19 facilities. Moreover, more HCWs in COVID-19 facilities reported other barriers to the use of PPE such as lower perceived risk of getting COVID-19, discomfort caused by some types of PPE, and that PPE interfere with patient-provider relationship, among others. Expectedly, we found that participants with high perceived effectiveness and those who received training on the appropriate use of PPE were more than 2 times more likely to be fully compliant with PPE. This result came to support the available evidence that higher perceived effectiveness of PPE and previous training predicted higher compliance.^13,14^ The most common barrier for appropriate use of PPE was the shortage of PPE, which is a global problem. The WHO has warned that shortage of PPE caused by increasing demand and misuse is putting the lives of HCWs at risk from SARS-CoV-2 infection and other infectious diseases.^29^ Therefore, to help countries in optimizing the rational use of PPE, the WHO issued a guidance for the rational use of PPE in healthcare settings and the effective management of supply chains.^30^ It is evident from previous outbreaks that PPEs are crucial to protect HCWs’ health and well-being and maintain a sustainable health workforce that can help in mitigating infectious outbreaks. We believe that adequate provision, clear guidance, and training on the appropriate use of PPE will increase HCWs’ confidence in delivering care. Primary Health Care Corporation worked in alignment with the Ministry of Public Health in Qatar and secondary care to develop an emergency action plan in response to the pandemic and to provide the necessary support for HCWs in the context of COVID-19. It issued guidance and instruction to HCWs on the rational use of PPE and the recommended types of PPE to be used by the staff and patients in different settings, locations, and during different patient interactions. Moreover, to minimize the risk of infection for both patients and HCWs, PHCC introduced remote virtual consultations using telemedicine and home delivery of medications, and put on hold some of its preventive programs such as cancer screening, while maintaining essential services such as vaccination clinics for children younger than 5 years, and walk-in clinic and urgent care service.^31^ Entrance to health centers and triaging patients relied on thermal screening and checking the status on Ehteraz app (a mandatory mobile public health application for people 18 years and older that aims to ensure that people could be alerted when they were in the vicinity of anyone infected or awaiting test results, as the status of Ehteraz app differentiates infected [had a positive COVID-19 polymerase chain reaction test result] and noninfected people).^32^ In addition, PHCC dedicated a team for case investigation and contact tracing of infected cases among PHCC staff, and also, HCWs under PHCC were among the first to receive COVID-19 vaccinations in Qatar. With the emergence and spread of new SARS-CoV-2 variants and the uncertainties surrounding the efficacy of the available vaccines against the new emerging variants, everyone and particularly HCWs should continue complying with the standard precautions including the appropriate use of PPE.

### Strengths and Limitations

This study has several strengths. First, it is one of the few studies in the Middle East to explore HCWs’ compliance with PPE during the current COVID-19 pandemic. Second, we managed to include an acceptable number of HCWs from the primary healthcare setting, which might increase our confidence in generalizing the results to the primary HCW population in Qatar. Although we provided some insights into the compliance of HCWs with PPE during this unprecedented crisis of COVID-19, we do acknowledge some limitations. First, the main limitation of this study that might introduce recall and social desirability bias is relying on the self-reporting by HCWs for collecting data and not on the direct observation of their practices. Hence, the detected compliance rates should be viewed cautiously. However, the only means for collecting data during COVID-19 in line with the national recommendations of maintaining physical distancing was using Web-based surveys. Second, institutional recommendations and instructions regarding the use of PPE might influence HCWs’ compliance and affect our results. Lastly, using the cross-sectional design of this study hindered us from establishing how compliance with PPE translates into lower incidence of COVID-19 infection among HCWs on the ground, which requires an alternative study design.

## CONCLUSIONS

Despite the high perceived effectiveness HCWs in primary healthcare settings had for PPE in protecting against COVID-19 infection, their full compliance rate with using PPE was moderate and needs further improvement. Healthcare workers’ age, nationality, health center region, area of work, clinical experience, frequency of interaction with suspected or confirmed COVID-19 cases, and the perceived effectiveness of PPE were significant predictors of full compliance with PPE. Shortage of PPE was the most commonly reported barrier. Continuous monitoring and conducting clinical audits and provision of adequate supplies of PPE are among the most important strategies to be followed by policy makers and managers of health centers to improve compliance rates among HCWs. Further research that involves direct observation of HCWs’ compliance with PPE is recommended, and investigating the impact of noncompliance on delivery of care and patient safety is needed.
